# Supplementary material for: microRNA Expression Profile in Obesity-Induced Kidney Disease Driven by High-Fat Diet in Mice
Source: Nutrients. 2024 Feb 28;16(5):691. doi: 10.3390/nu16050691 (PMC10934936; doi:10.3390/nu16050691)
Supplement: Supplementary file 1 [file nutrients-16-00691-s001.zip › Figures S1 and S2.pdf]

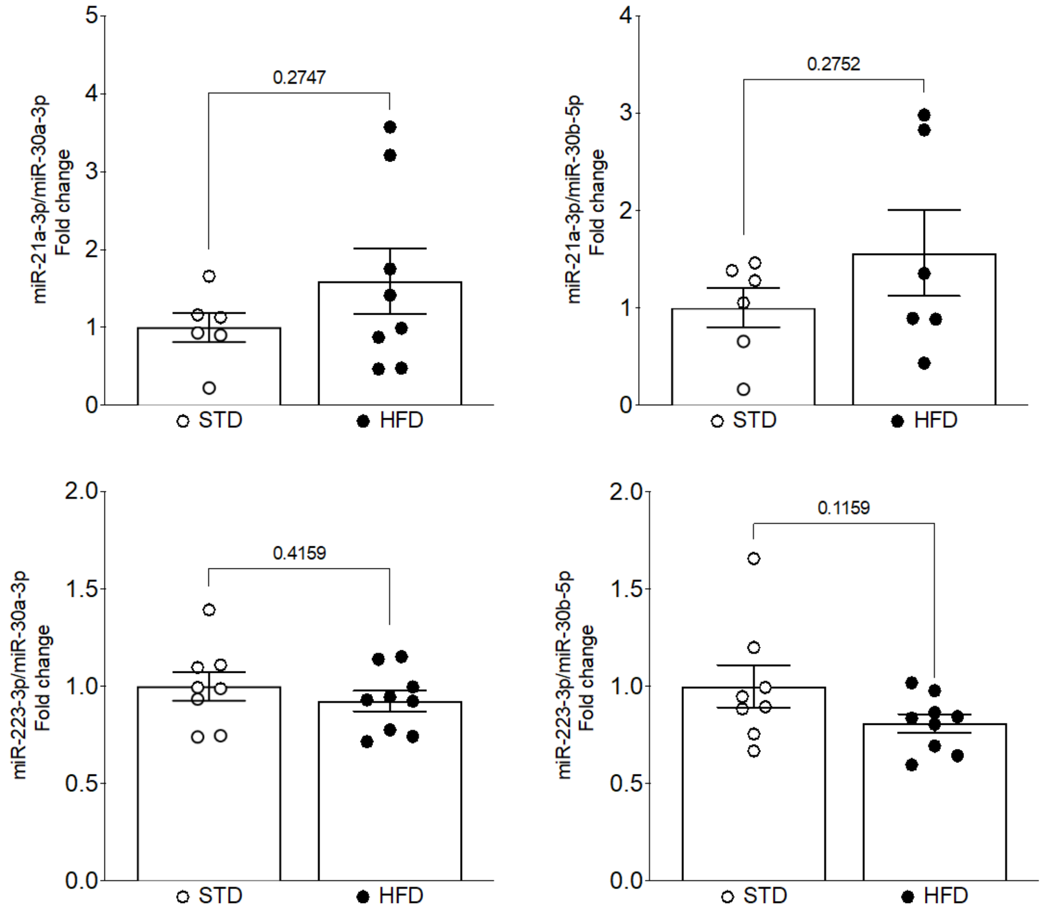

Figure S1. Expression levels of miR-21a-3p and miR-223-3p in the mouse kidney upon HFD feeding. miRNAs levels were determined by real-time qPCR and expressed as fold change over STD (value = 1.0) after normalizing for both miR-30a-3p and miR-30b-5p, as endogenous controls. Data are presented as mean ± SEM (n=6-9 mice/group).

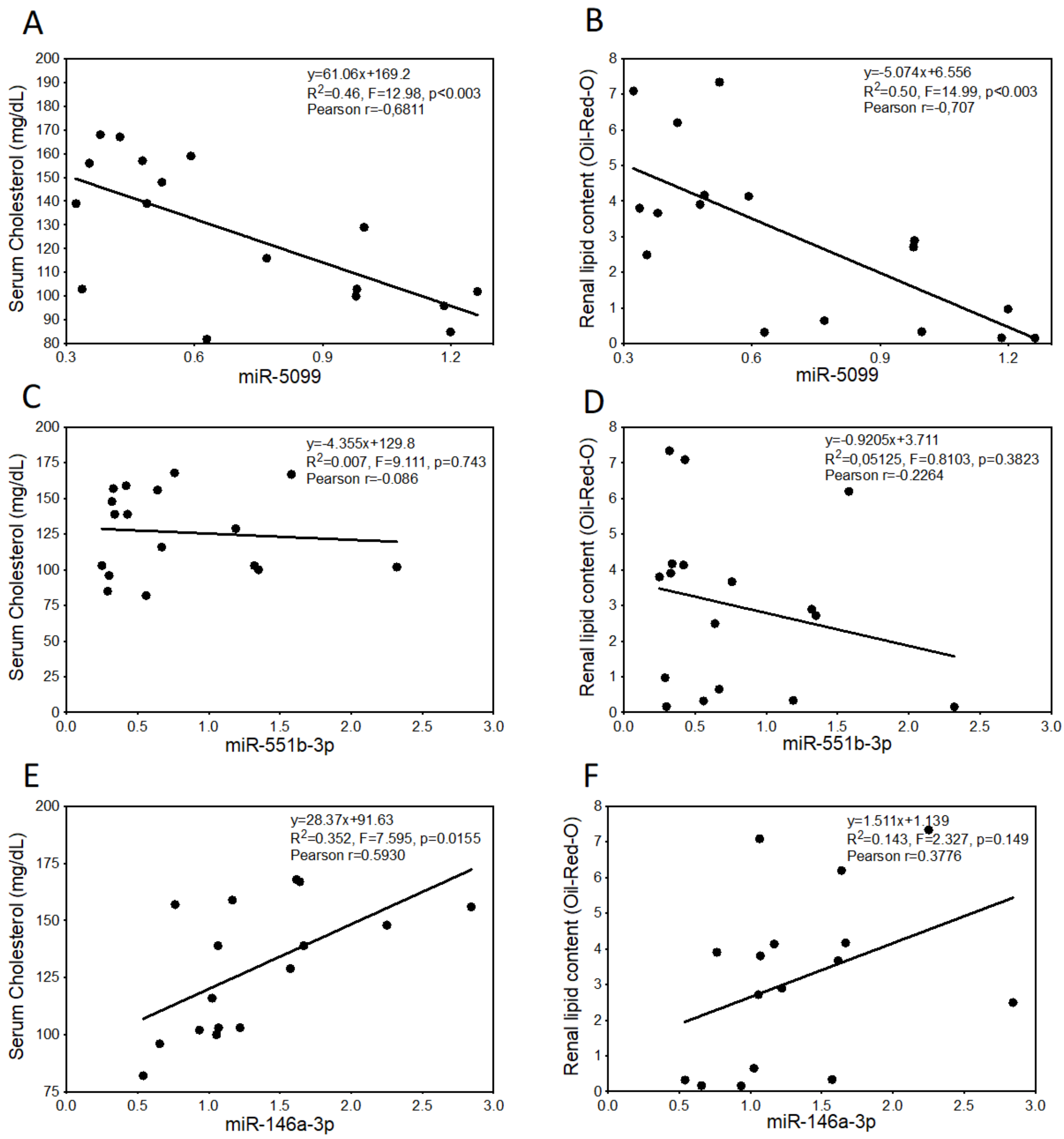

Figure S2. Correlation analysis between miR-5099, miR-551b-3p, miR-146a-3p expressions and serum cholesterol levels and/or renal lipid content. miRNAs levels were determined by real-time qPCR and expressed as fold change over STD (value = 1.0) after normalizing for endogenous controls. Serum cholesterol and renal lipid content were assessed by standard methods explained in the Material and Methods sections. A linear correlation analysis was performed between the miR-5099 and serum cholesterol (A); miR-5099 and renal lipid content (B); miR-551b-3p and serum cholesterol (C); miR-551b-3p and renal lipid content (D); miR-146-3p and serum cholesterol (E); miR-146-3p and renal lipid content (F); The Spearman correlation coefficient (R) and p value are shown.
